# MYC and HSF1 Cooperate to Drive Sensitivity to Polo-like Kinase 1 Inhibitor Volasertib in High-grade Serous Ovarian Cancer

**DOI:** 10.1158/2767-9764.CRC-24-0400

**Published:** 2025-02-06

**Authors:** Imade Williams, Matthew O’Malley, Haddie DeHart, Bobby Walker, Vrushabh Ulhaskumar, Pranav Jothirajah, Haimanti Ray, Lisa M. Landrum, Joe R. Delaney, Kenneth P. Nephew, Richard L. Carpenter

**Affiliations:** 1 Medical Sciences Program, Indiana University School of Medicine, Bloomington, Indiana.; 2 Melvin and Bren Simon Comprehensive Cancer Center, Indianapolis, Indiana.; 3 Obstetrics and Gynecology, Indiana University School of Medicine, Indianapolis, Indiana.; 4 Department of Biochemistry and Molecular Biology, Medical University of South Carolina, Charleston, South Carolina.; 5 Anatomy, Cell Biology and Physiology, Indiana University School of Medicine, Indianapolis, Indiana.; 6 Department of Biochemistry and Molecular Biology, Indiana University School of Medicine, Indianapolis, Indiana.

## Abstract

Ovarian cancer is a deadly gynecologic disease with frequent recurrence. Current treatments for patients include platinum-based therapy regimens with PARP inhibitors specific for homologous recombination–deficient high-grade serous ovarian cancers (HGSOC). Despite initial effectiveness, patients inevitably develop disease progression as tumor cells acquire resistance. Toward the development of new therapeutic avenues, we describe a gene amplification involving both heat shock factor 1 (*HSF1*) and *MYC*, wherein these two genes are co-amplified in more than 30% of patients with HGSOC. We further found that HSF1 and MYC transcriptional activities were highly correlated with human HGSOC tumors and cell lines, suggesting that they may cooperate in the disease. CUT&RUN sequencing for HSF1 and MYC revealed overlapping HSF1 and MYC binding throughout the genome. Moreover, the binding peaks of both transcription factors in HGSOC cells were nearly identical, and a protein–protein interaction between HSF1 and MYC was detected, supporting molecular cooperation. Supporting a functional cooperation of these two transcription factors, the growth of HGSOC cells with the co-amplification was dependent on both HSF1 and MYC. To identify a therapeutic target that could take advantage of this unique HSF1 and MYC dependency, polo-like kinase 1 (PLK1) was correlated with HSF1 and MYC in HGSOC specimens. Targeting PLK1 with volasertib revealed a greater than 200-fold increased potency in *HSF1*–*MYC* co-amplified HGSOC cells compared with those with wild-type *HSF1* and *MYC* copy numbers. Although the success of volasertib and other PLK1 inhibitors in clinical trials has been modest, the current study suggests that targeting PLK1 using a precision medicine approach based on *HSF1*–*MYC* co-amplification as a biomarker in HGSOC would improve therapy response and patient outcomes.

**Significance:**

We show that *HSF1* and *MYC* genes are co-amplified in more than 30% of HGSOC and demonstrate that HSF1 and MYC functionally cooperate to drive the growth of HGSOC cells. This work provides the foundation for *HSF1* and *MYC* co-amplification as a biomarker for treatment efficacy of the polo-like kinase 1 inhibitor volasertib in HGSOC.

## Introduction

Ovarian cancer is the fifth leading cause of cancer-related deaths in women in the United States and has a dismal prognosis (1) with high-grade serous ovarian cancer (HGSOC) subtype accounting for 70% to 80% of ovarian cancer deaths (2–4). HGSOC is a chemotherapy-responsive tumor with high initial response rates to standard therapy consisting of platinum (Pt) and paclitaxel. However, most women eventually develop recurrence and chemotherapy-resistant disease. Recurrent ovarian cancer is essentially incurable (5). There is an urgent need for novel and improved therapies for HGSOC (6). In particular, precision medicine approaches to identify subgroups of patients with HGSOC that have a higher likelihood of responding to any given therapy may improve patient outcomes.

Heat shock factor 1 (HSF1) is a transcription factor that was originally discovered as the master regulator of the heat shock response (7, 8). This physiologic role of HSF1 includes the transcriptional upregulation of chaperone HSPs in response to cellular stressors (9). HSF1 was identified to be more active in basal or triple-negative breast cancers (10), which have striking molecular similarities to HGSOC, and HSF1 was recently shown to be overexpressed and/or hyperactivated in HGSOC and other solid tumors (11–14).

The oncogenic transcription factor MYC has been reported to be overexpressed at the RNA level in up to 60% of ovarian tumors (15–17). In HGSOC, MYC has been shown to promote several mechanisms that induce oncogenesis and promote cancer progression, including proliferation and tumor cell metabolism (18). MYC expression was also demonstrated to be a prognostic marker for response to chemotherapy in patients with HGSOC (19, 20). There was a recent report that MYC can cooperate with HSF1 in non-cancer cells (21) and in hepatocellular carcinoma (HCC; ref. 22). However, whether they cooperate or interact in HGSOC remains to be established.

Polo-like kinase 1 (PLK1) is a serine/threonine kinase that has functions in cell-cycle progression, DNA damage response, and replication stress (23). PLK1 has been shown to be overexpressed and associated with poor patient outcomes in several human cancers, including HGSOC (24, 25). PLK1 is a therapeutic target, and compounds targeting PLK1 have been examined in clinical trials, including volasertib (BI-6727; ref. 23). Despite a clear therapeutic window for PLK1 inhibition, the efficacy of these compounds has been limited by treatment toxicities (23). However, PLK1 has been shown to directly phosphorylate both MYC and HSF1 to enhance their activity and protein stabilization (26–30). Toward a precision medicine approach that can identify patients who would benefit from PLK1 inhibition, the current study identified a therapeutic vulnerability with HSF1–MYC co-amplification in HGSOC. This co-amplification accounted for approximately one third of patients and has significance for other solid tumors, including breast cancer. We demonstrate that these two transcription factors have a functional interaction and cooperation in HGSOC cells. Furthermore, HGSOC cells with HSF1 and MYC co-amplification are more sensitive to PLK1 inhibition than those with normal copy numbers of HSF1 and MYC, suggesting that *HSF1* and *MYC* co-amplification could serve as a biomarker for treatment efficacy of the PLK1 inhibitors in patients with HGSOC.

## Materials and methods

### The Cancer Genome Atlas analysis

Gene amplification status in all The Cancer Genome Atlas (TCGA) cohorts was determined using called amplification status (≥2 copy-number gain) from publicly available TCGA data in cBioPortal.org. RNA sequencing of TCGA ovarian cancer (TCGA-OV) cohort was downloaded in reads per kilobase million (RPKM) for included analyses. MYC activity was assessed using a published gene signature (31) along with HSF1 activity (10).

### Cell culture and reagents

Unless otherwise indicated, all cell lines were purchased from ATCC and cultured in ATCC-recommended culture media at 37°C with 5% CO_2_. FTE-MYC (FT33-Tag-MYC) cells were a generous gift from Dr. Ronny Drapkin of the University of Pennsylvania, Philadelphia, PA (32). OvTrpMyc-F318LOV cells were generated from primary mouse tumors and described in our recent study (33). Cells are tested for *Mycoplasma* monthly. Cell lines were authenticated by short tandem repeat profiling annually. Reagents were purchased from Thermo Fisher Scientific unless otherwise noted. All siRNAs were purchased from Bioneer or Thermo Fisher Scientific, and sequences are listed in Supplementary Table S1. Volasertib was purchased from Cayman Chemical. pcDNA3-cmyc was a gift from Wafik El-Deiry (Addgene, plasmid #16011; RRID: Addgene_16011). FLAG-HSF1 was a gift from Stuart Calderwood (Addgene, plasmid #32537; RRID: Addgene_32537).

### Immunoblotting and immunoprecipitation

Immunoblotting and co-immunoprecipitation were performed as previously described (34). Antibodies used for immunoblotting and immunoprecipitation included MYC (Cell Signaling Technology, #13987S; RRID: AB_2631168), HSF1 (Cell Signaling Technology, #12972; RRID: AB_2798072), PLK1 (Cell Signaling Technology, #4513; RRID: AB_2167409), β-actin (Cell Signaling Technology, #3700; RRID: AB_2242334), GAPDH (Cell Signaling Technology, #2118; RRID: AB_561053), and p-HSF1 (S326; Abcam, #76076; RRID: AB_1310328).

### CUT&RUN sequencing

CUTANA CUT&RUN kit (EpiCypher) was used as previously described (21). Briefly, proliferating OVCAR8 cells were cross-linked with 1% formaldehyde in PBS for 1 minute on culture plates, followed by glycine quenching. The cells were then scraped and counted to 5 × 10^5^ cells and then incubated with IgG (Cell Signaling Technology, #3900S; RRID: AB_1550038), MYC (Cell Signaling Technology, #13987S; RRID: AB_2631168), or HSF1 (Cell Signaling Technology, #12972; RRID: AB_2798072) antibodies, according to the kit manufacturer’s instructions. Cross-link reversal was performed using 0.8 μL of 10% SDS and 1 μL of 10 μg/μL proteinase K and incubated at 55°C overnight. DNA was then purified for library generation and next-generation sequencing by the Center for Genomics and Bioinformatics at Indiana University. Libraries were prepared by NEBNext Ultra II DNA Library Prep Kit protocol (New England Biolabs, #E7645L) and analyzed by Agilent 4200 TapeStation. The libraries were pooled and loaded on a NextSeq 1000/2000 P2 Reagents (100 cycles) v3 flow cell (#20046811) configured to generate paired-end reads. The demultiplexing of the reads was performed using bcl2fastq, version 2.20.0. Raw data were trimmed and aligned to the human (GRCh38/hg38) reference genome using Bowtie 2 (35). CUT&RUN peaks were called using MACS2 (36) with a peak stringency set to 10^−4^ for HSF1 peaks and 10^−10^ for MYC peaks. Motif enrichment analysis was performed using MEME Suite AME (37), and gene binding tracks were visualized using Gviz (38). Gene Ontology was performed using Metascape (39).

### RT-qPCR

Total RNA was isolated using the Micro Total RNA isolation kit (Zymo Research). RNA was reverse-transcribed using random primers from a reverse transcription kit (Applied Biosystems). qPCR was performed using SYBR Green Universal master mix (Applied Biosystems) along with gene-specific primers using QuantStudio 3 (Applied Biosystems). Primers used are listed in Supplementary Table S2.

### IHC

Tissues were subjected to IHC as previously described by us (10). Tissues were purchased commercially from TissueArray.com (#OV8010a). Briefly, slides were deparaffinized, rehydrated prior to antigen retrieval using heat and pressure (2100 Antigen Retriever; Aptum Biologics), endogenous peroxidase activity blocked with Bloxall (Vector Laboratories, #SP-6000–100), and signal developed with 3, 3'-diaminobenzidine (DAB) (Vector Laboratories, #SK-4105). Antibodies used for IHC included MYC (Abcam, #ab32072; RRID: AB_731658), HSF1 (Cell Signaling Technology, #4356; RRID: AB_2120258), and p-PLK1 (T210; Abcam, #ab155095). Slides were imaged with Motic EasyScan and analyzed with QuPath (40).

### Cell viability and clonogenic growth

Cell viability assays were performed with CellTiter-Blue (Promega) according to the manufacturer’s instructions. Clonogenic growth assays were performed by seeding <1,000 cells into six-well plates and staining with crystal violet after 5 to 7 days of growth. Colonies were quantified using Fiji.

### Spheroids

Ovarian cancer cells (2,000) were seeded into 24-well ultralow attachment plates (Corning) and grown in serum-free spheroid media, as previously described (41). Spheroids were grown in the presence or absence of volasertib for 7 days. To quantify spheroids, images of each well were analyzed in ImageJ to measure the area of spheroids within each well. Biological replicates were averaged and normalized to the control group.

### Statistical analysis

All statistical tests were performed as two-tailed tests. For two-group comparisons, a Student *t* test was used. For multiple-group comparisons, ANOVA with a Tukey *post hoc* test was used. All laboratory experiments were completed with a minimum of three biological replicates (e.g., qPCR, luciferase assay, etc.).

### Data availability

Next-generation sequencing data generated in this study were deposited at Gene Expression Omnibus at GSE271226. Publicly available datasets used include TCGA cancer datasets that were available via the TCGA Data Portal with some analyses performed using cBioportal.org. The Cancer Cell Line Encyclopedia (CCLE) was accessed via DepMap.org. All other raw data generated in this study are available upon request from the corresponding author.

## Results

### MYC and HSF1 are frequently co-amplified in HGSOC

**Figure 1:**
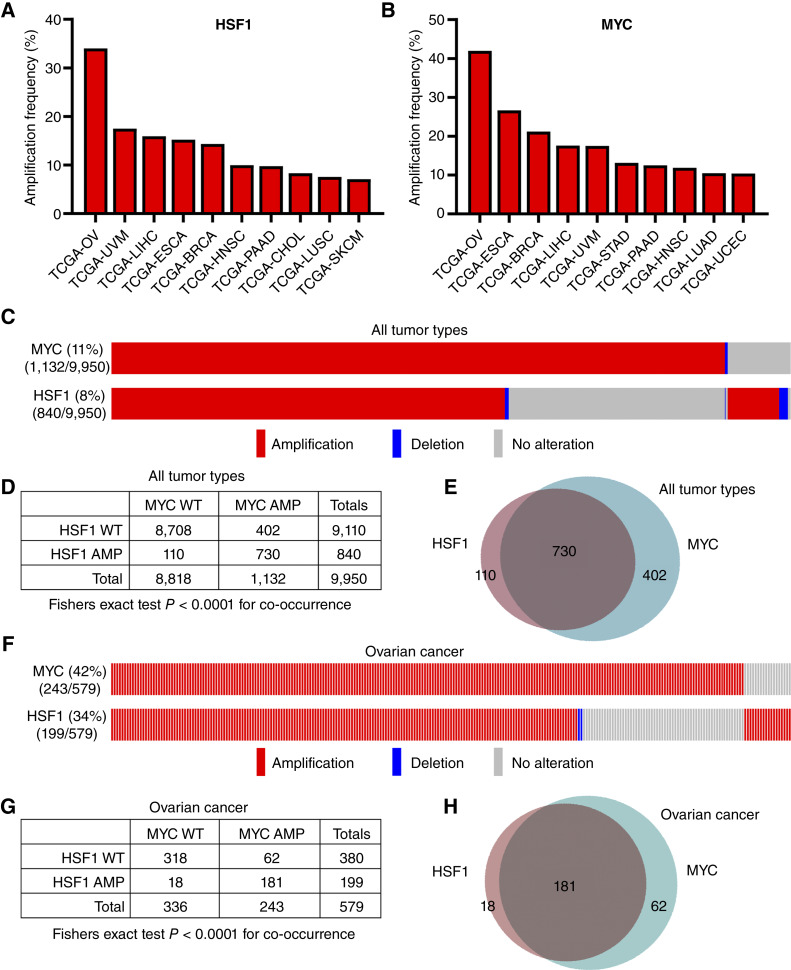
MYC and HSF1 are frequently co-amplified in HGSOC. Analysis of copy-number variation for HSF1 and MYC across cancer types using TCGA cohorts. Data were analyzed via cBioPortal. **A** and **B,** Amplification frequency for HSF1 (**A**) and MYC (**B**) across tumor types is presented, with amplification defined as ≥2 copy-number gains. **C,** Oncoprint for *HSF1* and *MYC* across all TCGA tumor types to indicate overlapping amplification for both genes. **D,** Table for *HSF1* and *MYC* amplification across all tumor types. A Fisher exact test was used to test the statistical significance for co-occurrence of amplification for both genes. **E,** Venn diagram of *HSF1* and *MYC* amplification for all tumor types indicating how many amplifications were overlapping. **F,** Oncoprint for HSF1 and MYC in TCGA-OV cohort to indicate overlapping amplifications in ovarian cancer. **G,** Table for *HSF1* and *MYC* amplification in ovarian cancer. A Fisher exact test was used to test the statistical significance for co-occurrence of amplification for both genes. **H,** Venn diagram of *HSF1* and *MYC* amplification in ovarian cancer indicating how many amplifications were overlapping. AMP, amplification.

Copy-number changes in the *HSF1* gene have previously been observed across tumor types (42, 43). Our analysis across TCGA cancer cohorts also observed several cancer types for which *HSF1* has significant copy-number gain, with the highest frequency of amplification (≥2 copy-number gain) in the cohort of patients with HGSOC (Fig. 1A). The *HSF1* gene is located on chromosome 8q (chr8q), which also contains the *MYC* gene. Interestingly, substantial copy-number gains of MYC were also observed across tumor types, with the most frequent gains seen in HGSOC tumors (Fig. 1B). Analysis of both *MYC* and *HSF1* copy numbers indicated that *MYC* had amplification in 11% (1,132/9,950) of human cancers, whereas *HSF1* was amplified in 8% (840/9,950) of cancers with an overlap in a substantial number of patients (Fig. 1C). In fact, greater than 7% (730/9,950) of human cancers had co-amplification of both *HSF1* and *MYC* genes (Fig. 1D and E), which was a statistically significant co-occurrence using a Fisher exact test (*P* < 0.0001).

Considering that both *HSF1* and *MYC* are located on chr8q, their co-amplification could be a random passenger amplification, if located on the same amplicon. Our analysis across tumor types showed that *MYC* amplification occurred without amplification of *HSF1* in a lower percentage of patients, whereas *HSF1* was also amplified without *MYC* amplification (Fig. 1D and E). These results suggest that *HSF1* and *MYC* can be separately amplified and not necessarily on the same amplicon. The frequency with which *MYC* and *HSF1* are co-amplified further indicates that co-amplification provides a growth or survival advantage for tumor cells, particularly for HGSOC compared with other tumor types, based on the high amplification frequency for *HSF1* and *MYC* in 34% (199/579) and 42% (243/579) of patients, respectively (Fig. 1F–H). There were patients with HGSOC with single amplification of *HSF1* or *MYC*, but their co-amplification was statistically significant for co-occurrence (Fisher exact test *P* < 0.0001; Fig. 1G). Expression of both HSF1 mRNA and MYC mRNA was strongly associated with the gene copy number (Supplementary Fig. S1A and S1B). The mRNA levels for both genes were highest in tumors with amplifications of each gene (Supplementary Fig. S1A and S1B), likely indicating a functional role. Interestingly, HSF1 mRNA levels were also elevated with *MYC* amplification, but MYC mRNA levels were unchanged with *HSF1* copy-number changes (Supplementary Fig. S1C and S1D). These results suggest that expression of *HSF1* and *MYC* is tied to their own gene copy number but not necessarily tied to copy-number changes for other chr8q genes, supporting that these genes may reside on the same chromosome but on separate amplicons.

### Transcriptional activities of MYC and HSF1 are correlated in HGSOC

**Figure 2:**
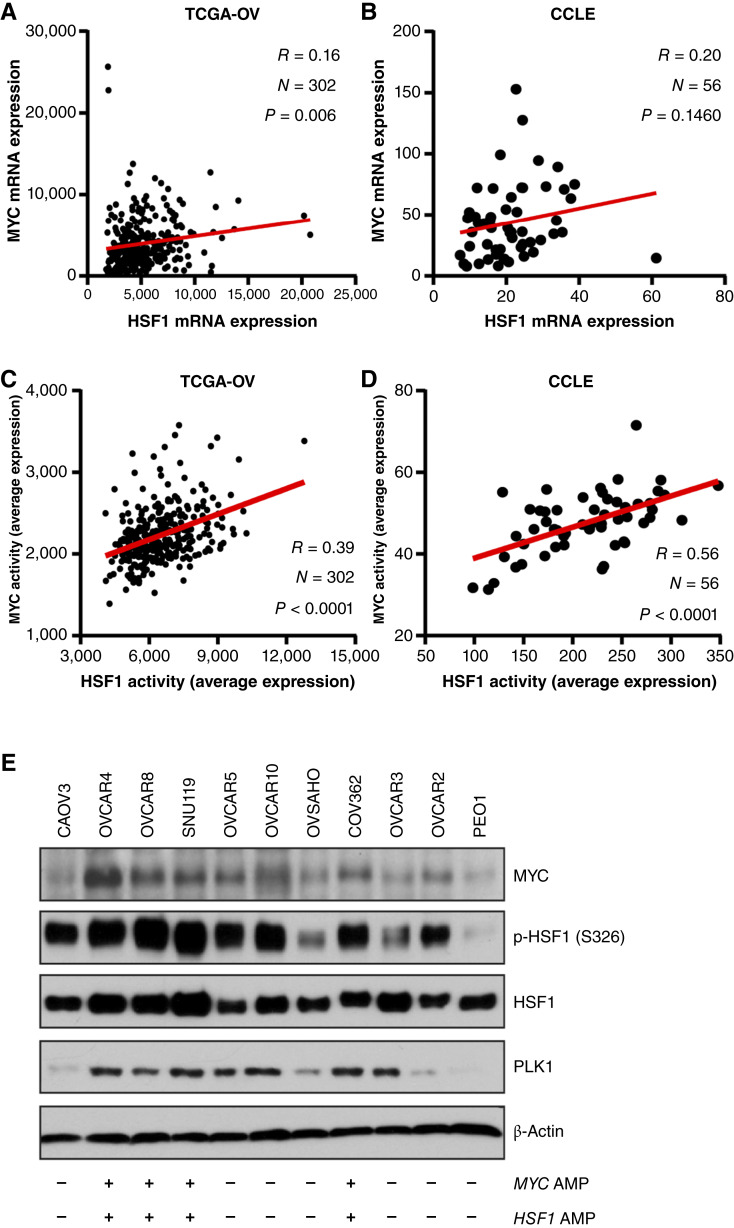
HSF1 activity is associated with MYC activity in HGSOC. **A** and **B,** HSF1 mRNA levels were plotted with MYC mRNA levels and analyzed with Pearson correlation in the TCGA-OV cohort (**A**) and HGSOC cell lines from the CCLE; (**B**). **C** and **D,** HSF1 and MYC transcriptional activities were calculated using published gene signatures and subjected to Pearson correlation using the TCGA-OV cohort (**C**) and HGSOC cell lines from the CCLE (**D**). **E,** Total protein from indicated cell lines were subjected to immunoblotting with the indicated antibodies. Immunoblotting was performed with three independent replicates. AMP, amplification.

Because *MYC* and *HSF1* have a high frequency of gene amplification in HGSOC, it was of interest to next investigate whether their expression is correlated. By examining the TCGA-OV cohort and HGSOC cell lines from the CCLE, expression of MYC was weakly associated with expression of HSF1 with correlation coefficients ranging from 0.16 to 0.20 and not reaching statistical significance in the CCLE (Fig. 2A and B). To next assess their transcriptional activities, we utilized published gene signatures for both MYC (31) and HSF1 (10), which are gene sets downstream of each transcription factor and the expression of which is directly correlated with MYC and HSF1 transcriptional activities. We found a strong positive correlation between MYC and HSF1 activities in the TCGA-OV cohort and HGSOC cell lines from the CCLE (Fig. 2C and D). We next assessed a panel of HGSOC cell lines and found that the amplification status of MYC and HSF1 was not perfectly predictive of their protein levels (Fig. 2E). We found no significant differences in MYC or HSF1 transcriptional activity based on copy number of either gene (Supplementary Fig. S2A–S2D), suggesting that copy-number gain does not necessarily correlate with increased transcriptional activity, despite copy-number gain affecting mRNA levels of each gene (Supplementary Fig. S2A and S2B). For both HSF1 and MYC, their expression correlated weakly with HSF1 activity in HGSOC (correlation coefficient 0.18–0.27; Supplementary Fig. S2E and S2F). The correlation between HSF1 and MYC transcriptional activities is likely to be functional because HSF1 and MYC copy-number gains did not correspond with transcriptional activity.

### Overlapping MYC and HSF1 gene targets in HGSOC cells

Considering the strong correlation between MYC and HSF1 transcriptional activities, we investigated whether they share similar gene targets, as suggested by a recent report on reduced global MYC DNA binding in the absence of HSF1 (21). We assessed genome binding patterns for both MYC and HSF1 by CUT&RUN sequencing. We selected the OVCAR8 cell line, in which both genes were amplified. The binding motifs for MYC and HSF1 were highly enriched in their respective CUT&RUN samples (Supplementary Fig. S3A and S3B). Furthermore, Gene Ontology for MYC- and HSF1-bound genes reported common ontologies associated with each transcription factor (Supplementary Fig. S3C and S3D). These results demonstrate that the binding profiles detected through this CUT&RUN are reflective of true binding patterns for MYC and HSF1.

**Figure 3:**
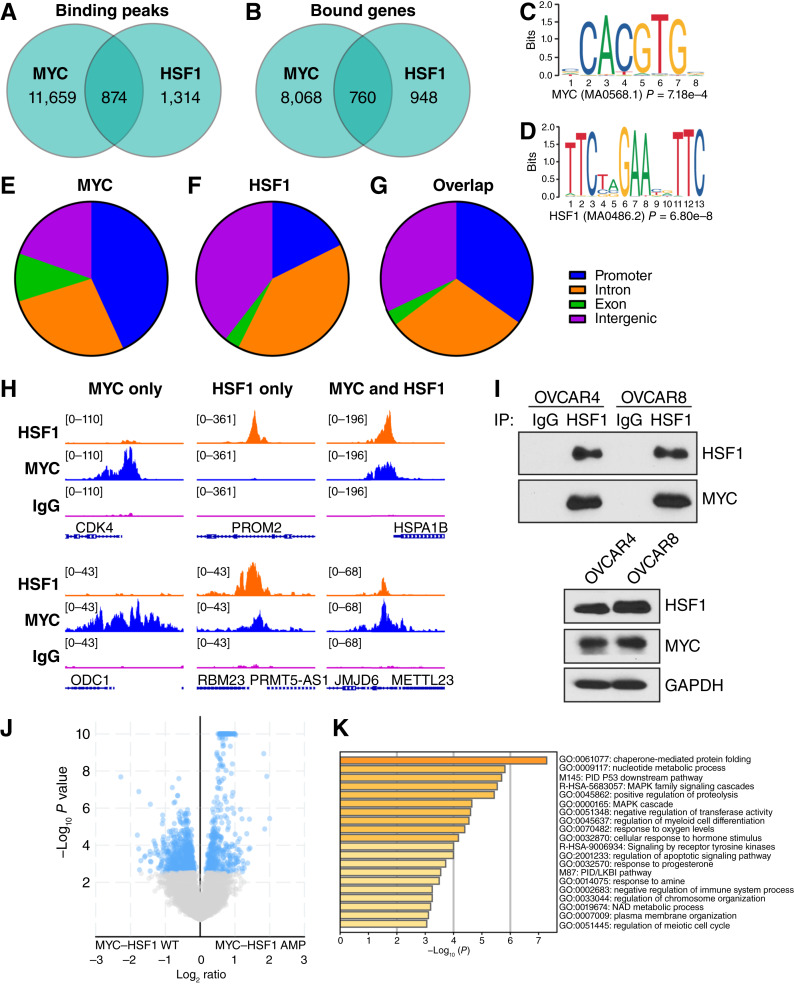
HSF1 and MYC share binding locations in the genome of OVCAR8 cells. OVCAR8 cells were subjected to CUT&RUN for both HSF1 and MYC. **A** and **B,** HSF1 and MYC called peaks (**A**) and annotated genes (**B**) are presented with the overlapping number between HSF1 and MYC in the Venn diagram overlap. **C** and **D,** Motif analysis showing the MYC-binding motif that presented in the HSF1 CUT&RUN (**C**) and the HSF1 binding motif that presented in the MYC CUT&RUN (**D**). **E–G,** Binding peaks for MYC (**E**), HSF1 (**F**), and the genes comprising the overlapping peaks (**G**) were classified by their binding location. **H,** Gene tracks from HSF1 and MYC CUT&RUN showing examples of genes with MYC-only binding (left), HSF1-only binding (middle), or genes with binding of both MYC and HSF1 (right). **I,** Total protein from OVCAR4 and OVCAR8 cells was subjected to immunoprecipitation with HSF1 antibodies and immunoblotted with the indicated antibodies. **J,** Volcano plot showing differentially expressed genes from TCGA-OV cohort comparing tumors with MYC–HSF1 co-amplification vs. tumors with a MYC–HSF1 WT copy number. **K,** Genes that were significantly higher expressed in MYC–HSF1 co-amplified tumors from **J** were overlapped with genes that were bound by both MYC and HSF1 (*n* = 83). These upregulated and MYC–HSF1–bound genes were subjected to Gene Ontology and enriched categories presented. AMP, amplification.

Across the genome, considerable MYC binding was observed (>12,000 peaks called), whereas more than 2,100 HSF1 peaks were detected (Fig. 3A). These peaks translated to >8,000 unique genes bound by MYC and >1,500 unique genes bound by HSF1 (Fig. 3B). There were 877 peaks that displayed an overlap in MYC and HSF1 binding, representing 39% of all HSF1 binding peaks but less than 7% of all MYC peaks because of the large number of MYC peaks detected (Fig. 3A). There were 760 unique genes that had overlapping peaks for both MYC and HSF1, representing ∼9% and 44% of genes bound by MYC and HSF1, respectively (Fig. 3B). There were an additional 392 genes wherein both MYC and HSF1 were bound, but peaks were not overlapping. Consistent with these results, the CUT&RUN results demonstrated that the MYC binding motif was significantly enriched in HSF1, and the HSF1 binding motif was significantly enriched in the MYC data (Fig. 3C and D).

With regard to the MYC-bound peaks, these were largely at intergenic regions, introns, and promoters with a lower percentage of bound sites located on exons (Fig. 3E). HSF1-bound peaks showed more binding to introns and intergenic regions with a lower number of bound sites at promoters (Fig. 3F). Focusing only on the MYC and HSF1 overlapping peaks, a more equal distribution of MYC and HSF1 binding across promoters, introns, and intergenic regions was observed (Fig. 3G). We also observed variations in binding between MYC and HSF1 across the genome (Fig. 3H). For example, there were genes bound only by MYC (*CDK4* and *ODC1*), genes bound only by HSF1 (*PROM2* and *RBM23*), and genes bound by both MYC and HSF1 (*HSPA1B* and *JMJD6*).

Based on the abundance of genes wherein MYC and HSF1 had overlapping peaks, we hypothesized that MYC and HSF1 form a protein complex. To test this, immunoprecipitation followed by immunoblotting was performed in two co-amplified HGSOC cell lines, OVCAR4 and OVCAR8. Immunoprecipitation of HSF1 in both cell lines resulted in the detection of MYC, demonstrating a protein–protein interaction in these cells (Fig. 3I). An interaction between HSF1 and MYC was also detected in OVCAR3 cells (Supplementary Fig. S4A) that have *MYC* and *HSF1* wild-type (WT) genes, consistent with previous work indicating an interaction between HSF1 and MYC in non-cancer cells (21). To investigate these MYC–HSF1–bound genes for potential functional significance, differential gene expression analysis was performed on the TCGA-OV cohort between samples with or without MYC–HSF1 co-amplification. We identified 4,155 differentially expressed genes between these two groups, with higher expression of 1,480 genes in MYC–HSF1 co-amplified tumors and higher expression of 2,675 genes in tumors that have a WT copy number of MYC and HSF1 (Fig. 3J). Of the 1,480 genes that were upregulated in MYC–HSF1 co-amplified tumors, 83 genes had overlapping MYC and HSF1 peaks. We next performed Gene Ontology using these 83 genes. Enriched categories with clear association with MYC and/or HSF1 functions were observed, such as chaperone-mediated folding, metabolism, and transcription (Fig. 3K). In addition, several other enriched categories that support tumor development and progression were seen, such as MAPK signaling, response to oxygen levels, and receptor tyrosine kinase signaling (Fig. 3K). Although this analysis is based on the gene amplification status of MYC and HSF1, we also completed a similar analysis based on MYC and HSF1 activities rather than copy-number status (Supplementary Fig. S4B and S4C). In this analysis, there were 87 genes that were significantly upregulated in tumors with high MYC and HSF1 activities that were also bound by MYC and HSF1 (Supplementary Fig. S4B). Gene Ontology of these 87 genes show similar categories as those described above relating to MYC and HSF1 functions (Supplementary Fig. S4C). These data suggest that amplified MYC and HSF1 are binding and upregulating genes that could support the initiation and progression of ovarian tumors.

### HSF1 and MYC functionally interact in HGSOC cells

**Figure 4:**
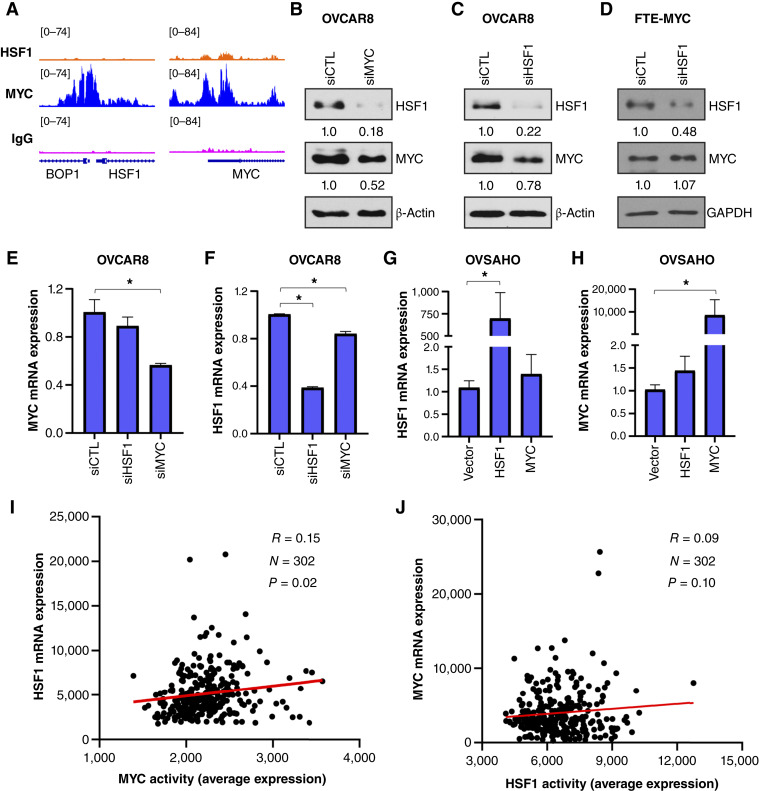
HSF1 and MYC cooperate in ovarian cancer cells. **A,** Analysis of HSF1 and MYC CUT&RUN showing gene tracks and binding at the *MYC* and *HSF1* genes. **B** and **C,** OVCAR8 cells were transfected with MYC (**B**) or HSF1 (**C**) siRNA for 48 hours. Total protein was subjected to immunoblotting. Bands were quantified by densitometry, and average changes are indicated under each band from at least two replicates. **D,** FTE-MYC cells were transfected with HSF1 siRNA for 48 hours. Total protein was subjected to immunoblotting. Bands were quantified by densitometry, and average changes are indicated under each band from at least two replicates. **E** and **F,** OVCAR8 cells were transfected with control, HSF1, or MYC siRNA for 48 hours. Total RNA was subjected to RT-qPCR for MYC (**E**) and HSF1 (**F**). **G** and **H,** OVSAHO cells were transfected with vector, HSF1, or MYC for 48 hours. Total RNA was subjected to RT-qPCR for HSF1 (**G**) and MYC (**H**). *, *P* < 0.05. siCTL, siRNA control. **I** and **J,** The TCGA-OV cohort was used to determine the correlation between MYC activity and HSF1 expression (**I**) as well as between HSF1 activity and MYC expression (**J**). Correlation was analyzed with Pearson correlation.

Analysis of the HSF1 and MYC CUT&RUN identified MYC binding to the *HSF1* promoter, as well as the *MYC* promoter, in OVCAR8 cells (Fig. 4A). Although there is a small peak for HSF1 binding at the MYC promoter, this is not robust and is likely no binding or weak binding (Fig. 4A). We hypothesized that this binding indicates that MYC and HSF1 may regulate each other. Knockdown of MYC lowered levels of HSF1 protein and knockdown of HSF1 resulted in a minor decrease in MYC protein levels in OVCAR8 cells (Fig. 4B and C; Supplementary Fig. S5A and S5B). Loss of HSF1 had no effect on MYC in human fallopian tube epithelial (FTE) cells, the cell of origin of HGSOC, which had been engineered to overexpress MYC (Fig. 4D; Supplementary Fig. S5C; ref. 32). This result was expected, as MYC is driven by a cytomegalovirus promoter in the FTE-MYC cells (32) but may also suggest that the protein interaction between MYC and HSF1 does not stabilize the MYC protein. Taken together, these data suggest that the effect of MYC on HSF1 expression is more pronounced than the reverse (the effect of HSF1 on MYC expression). We then confirmed this possibility at the RNA level. Knockdown of HSF1 had no effect on MYC RNA levels (Fig. 4E and F; Supplementary Fig. S5D and S5E). However, knockdown of MYC resulted in a modest but significant decrease in HSF1 expression (Fig. 4E and F), which was not replicated with a different set of siRNA (Supplementary Fig. S5D and S5E). Conversely, overexpression of MYC had no effect on HSF1 expression, and HSF1 overexpression did not alter MYC levels (Fig. 4G and H). Knockdown of either HSF1 or MYC in WT cells had no effect on the other (Supplementary Fig. S5F–S5I). These data indicate that MYC may have a minor effect on HSF1 expression in co-amplified cells. Consistent with these results, MYC activity in the TCGA-OV cohort was significantly associated with HSF1 expression (Fig. 4I), but HSF1 activity was not associated with MYC expression (Fig. 4J).

### HSF1–MYC co-amplification drives HGSOC cell growth

**Figure 5:**
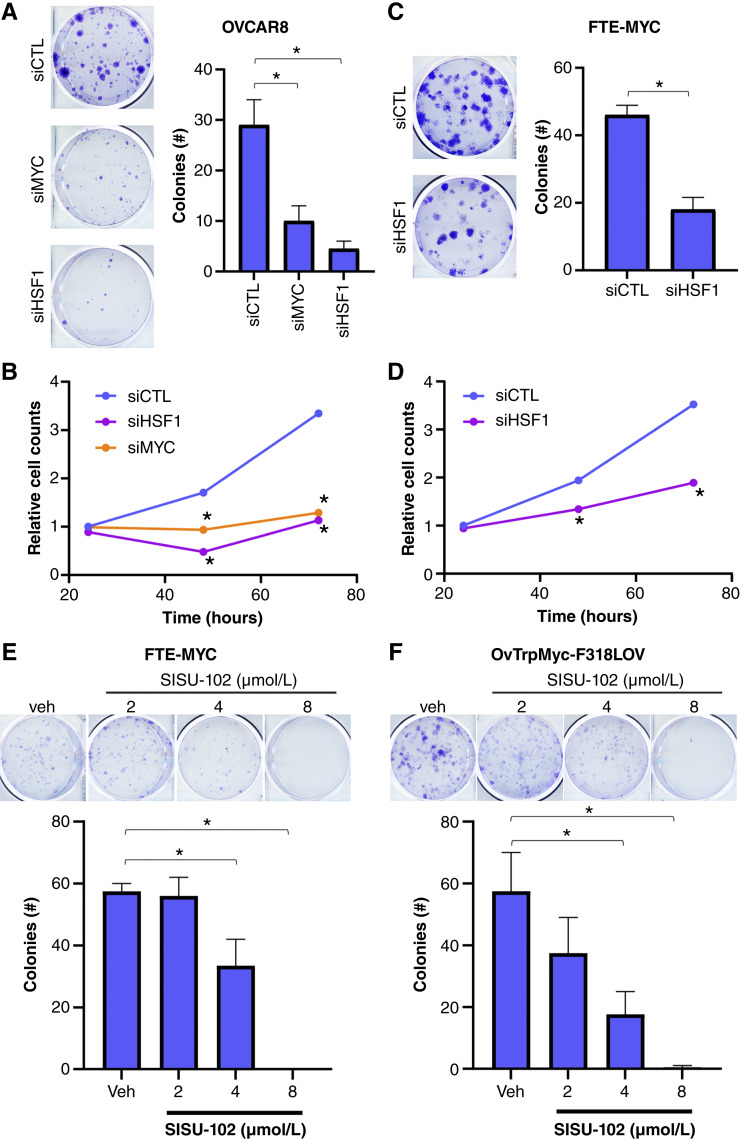
HSF1–MYC co-amplified HGSOC cells require both HSF1 and MYC for growth. **A** and **B,** OVCAR8 cells were transfected with control, HSF1, or MYC siRNA for 48 hours followed by a clonogenic growth assay for 7 days (**A**) or cell proliferation (**B**). **C** and **D,** FTE-MYC cells were transfected with control or HSF1 siRNA for 48 hours, followed by a clonogenic growth assay for 7 days (**C**) or cell proliferation (**D**). **E** and **F,** Either FTE-MYC (human; **E**) or OvTrpMyc-F318LOV (mouse) cells (**F**) were subjected to clonogenic growth assay for 7 days in the presence of vehicle or the HSF1 inhibitor SISU-102 at the indicated dosages. *, *P* < 0.05. siCTL, siRNA control; Veh, vehicle.

It was next of interest to investigate the effect of HSF1 and/or MYC amplification on HGSOC cell growth. Loss of either HSF1 or MYC in OVCAR8 cells significantly reduced clonogenic growth (Fig. 5A; Supplementary Fig. S6A) and cell proliferation (Fig. 5B; Supplementary Fig. S6B). Similarly, loss of HSF1 in FTE-MYC cells (32) significantly reduced clonogenic growth (Fig. 5C, Supplementary Fig. S6C) and proliferation (Fig. 5D; Supplementary Fig. S6D). OVCAR10 cells are WT for the *HSF1* and *MYC* genes and were also subjected to HSF1 or MYC knockdown. Colony formation in these WT cells was also reduced by ∼20% (Supplementary Fig. S6E), whereas the co-amplified cells were reduced by ∼80%. We further tested the dependency of FTE-MYC cells (32) and OvTrpMyc-F318LOV, which are MYC and mutant p53-driven mouse HGSOC cells (33), on HSF1 by using the HSF1-specific inhibitor SISU-102 (DTHIB; ref. 44). Treatment with SISU-102 inhibited clonogenic growth of both FTE-MYC (human) and OvTrpMyc-F318LOV (mouse) cells (Fig. 5E and F). Although the SISU-102 significantly reduced colony formation of these MYC-driven cells, it did not have any significant effect on OVCAR10 cells (WT for *HSF1* and *MYC* genes; Supplementary Fig. S7A). We further assessed how SISU-102 affected HSF1 and MYC levels in OVCAR8 co-amplified cells and found a dose-dependent reduction in HSF1 protein and a decrease in MYC protein at 8 μmol/L (Supplementary Fig. S7B). When HSF1 protein was rescued using an exogenous plasmid, SISU-102 only reduced HSF1 levels at the 8 μmol/L dose, at which MYC also decreased (Supplementary Fig. S7C). These data demonstrate that ovarian cancer cells with MYC–HSF1 co-amplification are highly sensitive to loss of either HSF1 or MYC, supporting a model whereby cells are dependent on both transcription factors. Additionally, inhibition of HSF1 led to a reduction in MYC protein levels that likely caused the reduction in growth of MYC-driven cells.

### MYC and HSF1 are associated with PLK1 in HGSOC

**Figure 6:**
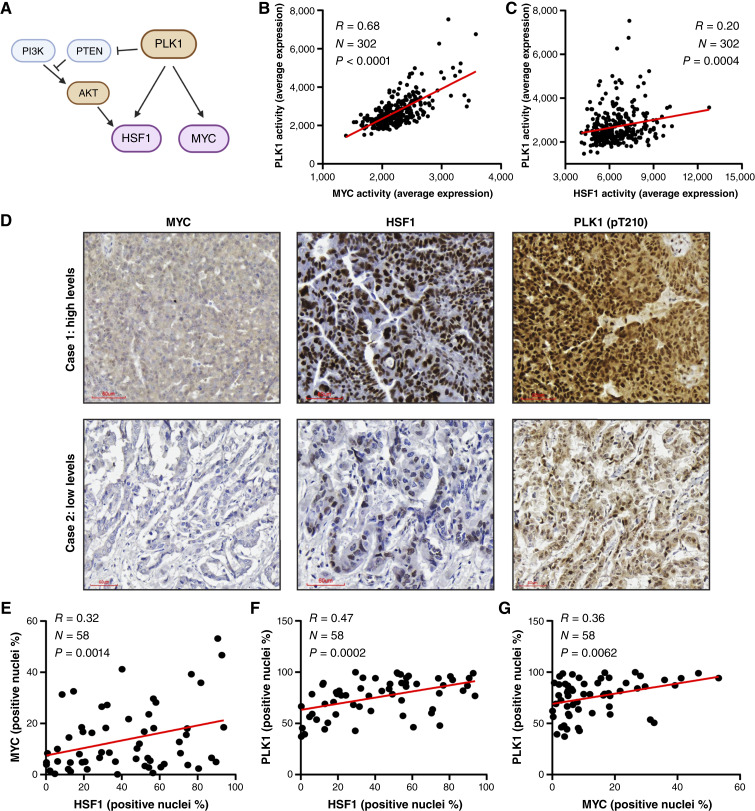
HSF1 and MYC are correlated with active PLK1 in HGSOC tumors. **A,** Diagram of the relationship between PLK1 and MYC/HSF1, indicating that PLK1 can directly regulate MYC and HSF1 through phosphorylation but also indirectly by regulating them through the PI3K–AKT pathway, among others. **B** and **C,** PLK1 activity was assessed using a published gene signature in the TCGA-OV cohort and correlated with activity signatures for MYC (**B**) or HSF1 (**C**). **D,** 58 HGSOC tumors were subjected to IHC with antibodies for MYC, HSF1, and active PLK1 (pT210). **E–G,** IHC in **D** was analyzed with QuPath to identify the percent of cells that have positive nuclei for these markers to indicate active levels. Pearson correlation was used to correlate active HSF1 and MYC (**E**), HSF1 and PLK1 (**F**), and MYC and PLK1 (**G**).

To identify possible therapeutic approaches that would benefit HGSOC tumors with *MYC* and *HSF1* co-amplification, PLK1 was identified as a kinase that can directly and indirectly regulate both MYC and HSF1 (26–30). MYC has also been previously shown to bind the *PLK1* gene (45), which we also observed in OVCAR8 cells (Supplementary Fig. S8A). Consequently, PLK1 could be an attractive therapeutic target to abolish this cancer-promoting signaling node (Fig. 6A). To test this, we first assessed the correlation of MYC and HSF1 activities with PLK1 activity in the TCGA-OV cohort using a published gene signature for PLK1 (46). PLK1 activity was significantly associated with both MYC and HSF1 activities (Fig. 6B and C). To further assess whether active PLK1 was associated with MYC or HSF1 in primary samples of patient with HGSOC, we performed IHC for active PLK1 (pT210), HSF1, and MYC using 58 tumor samples of patients with HGSOC (Fig. 6D). Nuclear levels of MYC and HSF1 were correlated (Fig. 6E), confirming the activity of the two transcription factors in the same tumors. Active PLK1 correlated with nuclear levels of both HSF1 and MYC (Fig. 6F and G), indicating a positive association between active PLK1 and MYC and HSF1. PLK1 protein levels in ovarian cancer cells also seem to correlate with MYC and HSF1 levels (Fig. 2E). These observations support that PLK1 could be an attractive therapeutic target to inhibit a key cancer-promoting signaling node.

### PLK1 inhibition is more effective with MYC and HSF1 dual amplification

**Figure 7:**
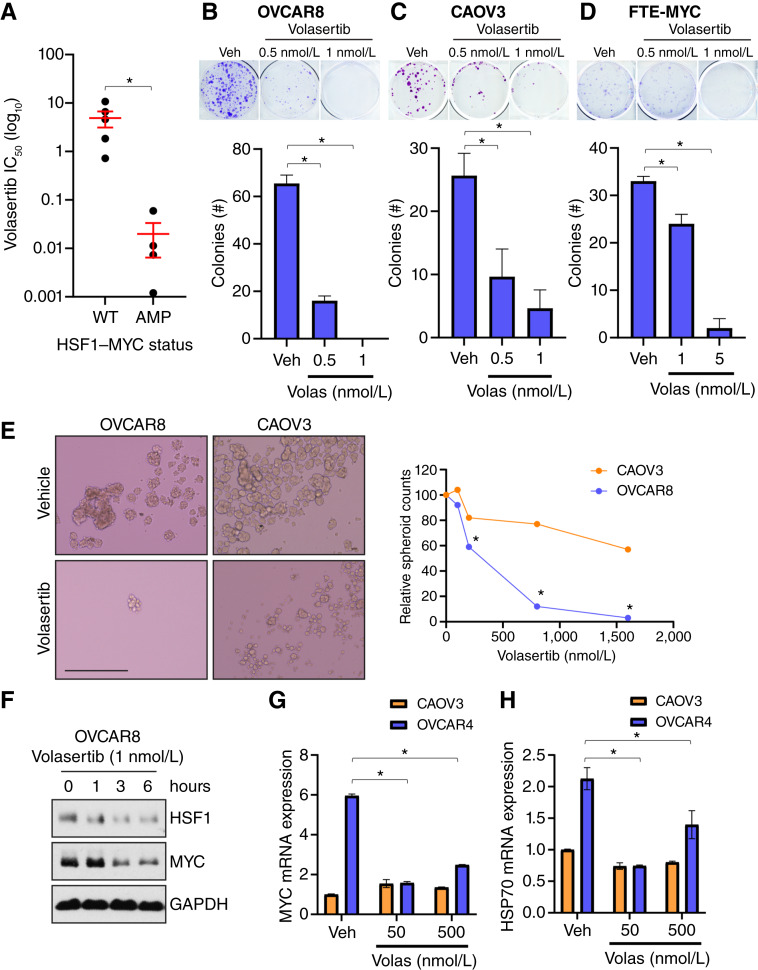
HSF1–MYC co-amplified HGSOC cells are highly sensitive to PLK1 inhibition with volasertib. **A,** IC_50_ for volasertib in HGSOC cells with HSF1 and MYC amplified or WT. **B–D,** OVCAR8 (**B**, AMP), CAOV3 (**C**, WT), or FTE-MYC (**D**) cells were subjected to a clonogenic growth assay for 7 days in the presence of vehicle or volasertib at indicated doses. **E,** OVCAR8 and CAOV3 cells were subjected to tumor spheroid growth for 12 days in the presence of vehicle or volasertib at the indicated doses. **F,** OVCAR8 cells were grown in the presence of vehicle or volasertib (1 nmol/L) for the indicated time periods. Total protein was subjected to immunoblotting for the indicated antibodies. **G** and **H,** OVCAR4 (AMP) or CAOV3 (WT) cells were treated with vehicle or volasertib at the indicated doses for 24 hours. Total RNA was subjected to RT-qPCR for MYC (**G**) or HSP70 (**H**). *, *P* < 0.05. AMP, amplification; Veh, vehicle; Volas, volasertib.

PLK1 is an active therapeutic target with several compounds targeting this kinase in clinical trials. In particular, volasertib (BI-6727) is a selective PLK1 inhibitor that has shown promise as an effective cancer therapy in early-phase clinical trials (47, 48). To assess whether volasertib has specificity for *MYC*–*HSF1* co-amplified HGSOC cells, we first determined the IC_50_ for volasertib in cell lines with or without *HSF1*–*MYC* co-amplification. The average IC_50_ for volasertib in *HSF1*–*MYC* co-amplified cell lines was 19.8 nmol/L, whereas cells that were WT for *HSF1* and *MYC* had an average IC_50_ of 4.9 μmol/L, a >200-fold difference (Fig. 7A). Volasertib also showed a greater reduction in clonogenic growth for *MYC*–*HSF1* co-amplified cells compared with *MYC*–*HSF1* WT cells (Fig. 7B and C). Consistent with genetic depletion or inhibition of HSF1, volasertib significantly reduced clonogenic growth of FTE-MYC cells (Fig. 7D). Furthermore, volasertib significantly reduced the ability of *HSF1*–*MYC* co-amplified OVCAR8 and OVCAR4 cells to form spheroids under low attachment conditions (Fig. 7E; Supplementary Fig. S8B), whereas volasertib had less effect on spheroid growth of *MYC*–*HSF1* WT CAOV3 and PEO1 cells (Fig. 7E; Supplementary Fig. S8B).

As PLK1 phosphorylation has been reported to increase the protein stability of MYC and HSF1 (26–30), we tested the effect of volasertib on MYC or HSF1 protein levels in co-amplified OVCAR8 cells. A time course of volasertib exposure indicated a loss of MYC and HSF1 proteins within 1 to 3 hours of 1 nmol/L volasertib exposure (Fig. 7F). Similarly, volasertib was also found to suppress expression of MYC and HSP70, a direct target of HSF1, in *MYC*–*HSF1* co-amplified OVCAR4 cells but not in CAOV3 cells that have WT *MYC* and *HSF1* copy numbers (Fig. 7G and H). It is worth noting that although HSP70 is a commonly used direct target of HSF1 to represent HSF1 activity, it certainly can be targeted by other transcription factors. Taken together, these results demonstrate that volasertib suppresses PLK1-mediated protein stabilization of HSF1 and MYC. Furthermore, inhibition of PLK1 can destabilize the HSF1 and MYC signaling node in HGSOC cells, indicating that *HSF1*–*MYC* co-amplification could serve as a potential biomarker for therapeutic response to PLK1 inhibition.

## Discussion

HGSOC is characterized by a high initial response to Pt-based therapies followed by recurrence and subsequent development of Pt resistance, which is universally fatal. Pt-based therapy regimens are commonly used in patients with HGSOC, regardless of the underlying molecular nature of the tumor. In this study, we describe a biomarker for sensitivity to PLK1 inhibition, based on increased copy number on chromosome 8, leading to gene amplification of both *HSF1* and *MYC*. Although PLK1 inhibitors have not met expectations in clinical trials, we suggest that targeting PLK1 using a precision medicine approach, one based on using *HSF1*–*MYC* co-amplification as a biomarker, would improve therapy response and patient outcomes. These data further demonstrate that *HSF1*–*MYC* co-amplification is present in several different cancer types but with specific enrichment in HGSOC, for reasons unknown at this point. In addition, the results of the current study support *HSF1*–*MYC* co-amplification as a potential biomarker for many cancer types and approximately one third of patients with HGSOC. Considering that volasertib directly targets PLK1, it could be argued that PLK1 expression could be a better predictor for volasertib response. However, sequencing for copy-number changes is routine for clinical assessment of ovarian tumors, making *HSF1*–*MYC* co-amplification more easily accessible as a clinical biomarker for HGSOC. PLK1 inhibitors may be more effective in treating patients with this co-amplification, particularly patients with HGSOC, warranting further clinical evaluation. Future studies will further test this concept *in vivo*, especially considering that genes co-regulated by MYC and HSF1 showed some indications that could only be relevant *in vivo*, such as metabolism and hypoxia.

PLK1 has been considered a viable therapeutic target in cancer for many years (49–51). Volasertib (BI-6727) is a selective PLK1 inhibitor that has shown promise as an effective cancer therapy in early-phase clinical trials with side effects being reversible and manageable (47, 48). However, phase II trials were disappointing with only modest antitumor activity as a monotherapy (47, 52). One of these phase II trials was conducted on patients with advanced ovarian cancer and showed that the median progression-free survival (PFS) for volasertib (13.1 weeks) was worse than that for chemotherapy (20.6 weeks). However, there were six patients in the study receiving volasertib that achieved a PFS of more than 1 year, whereas no patients receiving chemotherapy achieved a PFS greater than 1 year (47). Additionally, patients receiving chemotherapy discontinued treatment because of adverse events more frequently than volasertib (47). Although the study did not perform any evaluation or analyses related to potential biomarkers that could delineate these patients who responded well to volasertib, the current study would suggest that *HSF1*–*MYC* co-amplification could potentially serve as a biomarker for patients that would respond more favorably to volasertib or other PLK1 inhibitors. Thus, to improve response to PLK1 inhibition, we suggest a precision medicine approach based on tumors harboring co-amplification of these two transcription factors. These studies support this idea of using co-amplification as a biomarker, but mechanistically whether it is required for both *MYC* and *HSF1* genes to be amplified for this sensitivity is unclear. We primarily analyzed co-amplified cells because there are no ovarian cancer cell lines with only amplification of one of these genes. Considering that MYC was seen to bind the *HSF1* gene promoter, overexpressing only MYC may still lead to co-overexpression, which was evident in the FTE-MYC cells used in these studies. Studies are ongoing to engineer a system in which these can be tested individually in ovarian cancer cells.

The current study also adds to the ongoing discoveries indicating biological and physical interactions between HSF1 and MYC. MYC is a frequent oncogenic driver across many tumor types. It has previously been reported that MYC-driven HCC requires HSF1 for tumor formation (22), and results from the current study support that this dependency also occurs in HGSOC. We also observed HSF1 and MYC co-amplification in TCGA HCC data but in a lower percentage of patients compared with patients with HGSOC. A recent elegant study demonstrated that HSF1 potentiates MYC transcriptional activity driven by a physical interaction between the transcription factors and that HSF1 was essential to recruitment of epigenetic machinery required for gene regulation (21). Although these studies were not in cancer cells, the current results support that MYC and HSF1 also form a protein complex in ovarian cancer cells and MYC-driven ovarian cancer cells are dependent on HSF1. The current study also found a significant number of binding sites for HSF1 and MYC in ovarian cancer cells wherein the binding peaks are overlapping, which would be consistent with previous reports suggesting that HSF1 and MYC form protein complexes with DNA (21). The MYC and HSF1 interaction will be the subject of future investigations, as this interaction is likely to be critical to tumorigenesis and perhaps several other functions known to be driven by MYC and HSF1, such as the cancer stem–like population among others (14, 53).

The current study is the first to show genome binding patterns for both HSF1 and MYC in co-amplified ovarian cancer cells. These data further support a gene-regulating protein complex involving both HSF1 and MYC, evidenced by highly similar binding peaks at many gene targets. These data also indicate a MYC–HSF1 complex that can bind both proximal promoters and distal enhancers, consistent with previous observations that these transcription factors can function at both promoter and enhancer locations in cancer cells (54, 55). Interestingly, we also found that both the *MYC* and *HSF1* genes are themselves targets. We observed MYC binding the *HSF1* and *MYC* promoters, indicating at least some dependency of HSF1 expression on MYC. This may be in part due to the high HSF1 copy number because a previous study showed that HSF1 expression has a greater dependency on MYC in non-cancer cells (21). Additional targets of the HSF1 and MYC complex could offer some insights to their functions in ovarian tumors. For example, the estrogen-related receptor α (*ESRRA*) gene had overlapping binding of HSF1 and MYC and had increased expression in co-amplified tumors. Estrogen-related receptor α has previously been shown to play a role in ovarian tumors through the regulation of metabolism and mitochondria (56, 57). This could be a method by which HSF1 and MYC affect metabolism to support their functions in tumor cells. Additionally, the long noncoding RNA *NEAT1* was also a target of both HSF1 and MYC. In addition to a direct role in ovarian cancer for NEAT1 by affecting homologous recombination (58), NEAT1 is also involved in phase-separated transcriptional condensates (59). Recent work has shown that HSF1 participates in transcriptional condensates to enhance the efficiency of HSF1 activity (60, 61). HSF1 and MYC were also bound to the *MED15* gene, which also participates in transcriptional condensates (62). This could point to HSF1 and MYC enhancing expression of several components that will promote the formation of transcriptional condensates that enhance their activity and efficiency. Future studies will ascertain the importance of the physical interaction between HSF1 and MYC by mapping the necessary regions for the interaction with the goal of blocking the physical interaction without compromising protein levels.

The specific vulnerability of HSF1–MYC co-amplified cells to PLK1 inhibition seems to originate from the signaling node relating HSF1 and MYC to other growth-promoting pathways. In addition to directly phosphorylating both MYC and HSF1, PLK1 can also indirectly affect MYC and HSF1 function through phosphorylation and deactivation of PTEN, thereby enhancing the activity of PI3K-mediated signaling and leading to AKT and mTOR activation (26, 28, 29). Both AKT and mTOR activation has been shown to activate HSF1, whereas mTOR can also phosphorylate MYC (63–65). PLK1 can also inactivate the E3 ligase FBXW7 (45), which has been shown to downregulate both MYC and HSF1 (66). FBXW7 additionally suppresses several other growth-promoting pathways, including cyclin E and NOTCH1 (67). The current study indicates that MYC can also directly bind the *PLK1* promoter. We further suggest that the signaling network, through a positive feedback cycle, can reinforce its own activity, leading to increased proliferation. In this scenario, by providing cancer cells with a clear growth advantage by activating this signaling network and enabling efficient proliferation and suppression of growth-arresting pathways, the MYC–PLK1 interaction could play a critical role in tumor initiation.

Along with the current study, the link between HSF1 and MYC has been seen in cancer cells (22) and non-cancer cells (21), suggesting that their interaction is physiologic but can be co-opted in cancer cells. MYC has previously been shown to cooperate with the unfolded protein response through ATF4 (68). MYC cooperation with HSF1 may indicate that MYC cooperates with several stress response and proteostasis pathways, possibly because of the positive effects of MYC on protein translation. Several attempts to therapeutically target MYC or HSF1 have all largely failed to reach clinical trials. There is currently an ongoing phase Ia trial for the compound NXP800 (NCT05226507; ref. 69), which was initially labeled as an HSF1 pathway inhibitor but has since been reclassified as a GCN2 agonist. NXP800 was shown to inhibit HSF1 activity, but this seems to be an indirect effect on HSF1. The compound SISU-102 (DTHIB; ref. 44) is a direct inhibitor of HSF1 that is also currently under development at Sisu Pharmaceuticals. Future studies to further clarify the interaction of these two important transcriptional pathways in HSF1 and MYC, identify actionable therapeutic targets, and disrupt cancer-promoting pathways that could lead to clinical advances in a precision medicine therapeutic approach are warranted.

## Supplementary Material

Supplementary Figures and Legends This document includes all Supplementary Figures with their associated Figure legends.

Supplementary Tables This document contains all Supplementary Tables.
