# Supplementary material for: MYC and HSF1 Cooperate to Drive Sensitivity to Polo-like Kinase 1 Inhibitor Volasertib in High-grade Serous Ovarian Cancer
Source: Cancer Res Commun. 2025 Feb 6;5(2):253–66. doi: 10.1158/2767-9764.CRC-24-0400 (PMC11799878; doi:10.1158/2767-9764.CRC-24-0400)
Supplement: Supplementary Figures and Legends — This document includes all Supplementary Figures with their associated Figure legends. [file crc-24-0400_supplementary_figures_and_legends_suppsfl.docx]

**Supplemental Figures and Legends**

**
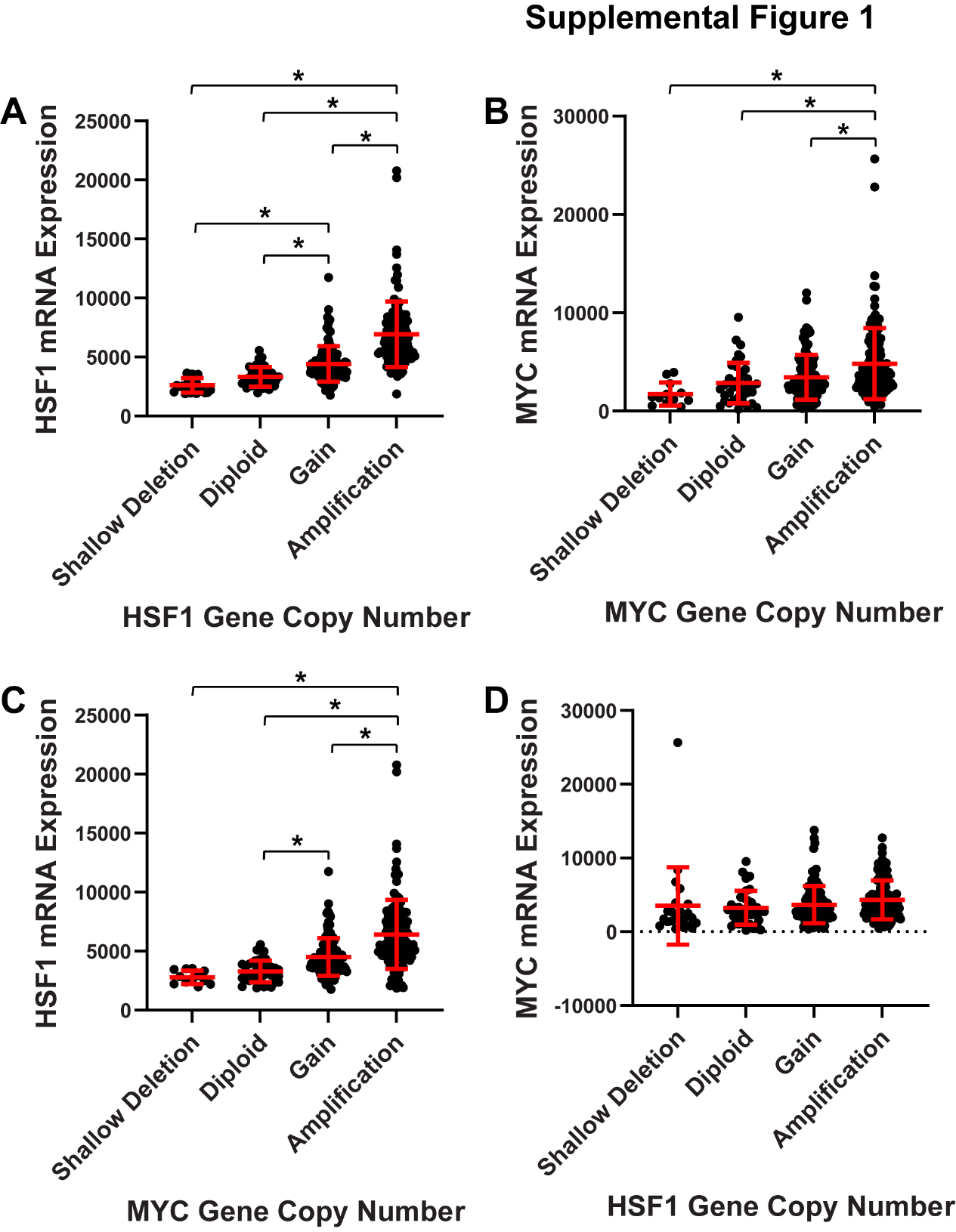
**

**Supplemental Figure 1: Effect of HSF1 or MYC copy number on mRNA expression.** The TCGA-OV cohort was used for this analysis and performed using cBioportal.org. A-B) Expression of HSF1 (A) and MYC (B) are presented in relation to their respective copy number changes. C-D) Expression of HSF1 as it relates to MYC copy number (C) and expression of MYC as it relates to HSF1 copy number (D). *Indicates p<0.01.

**
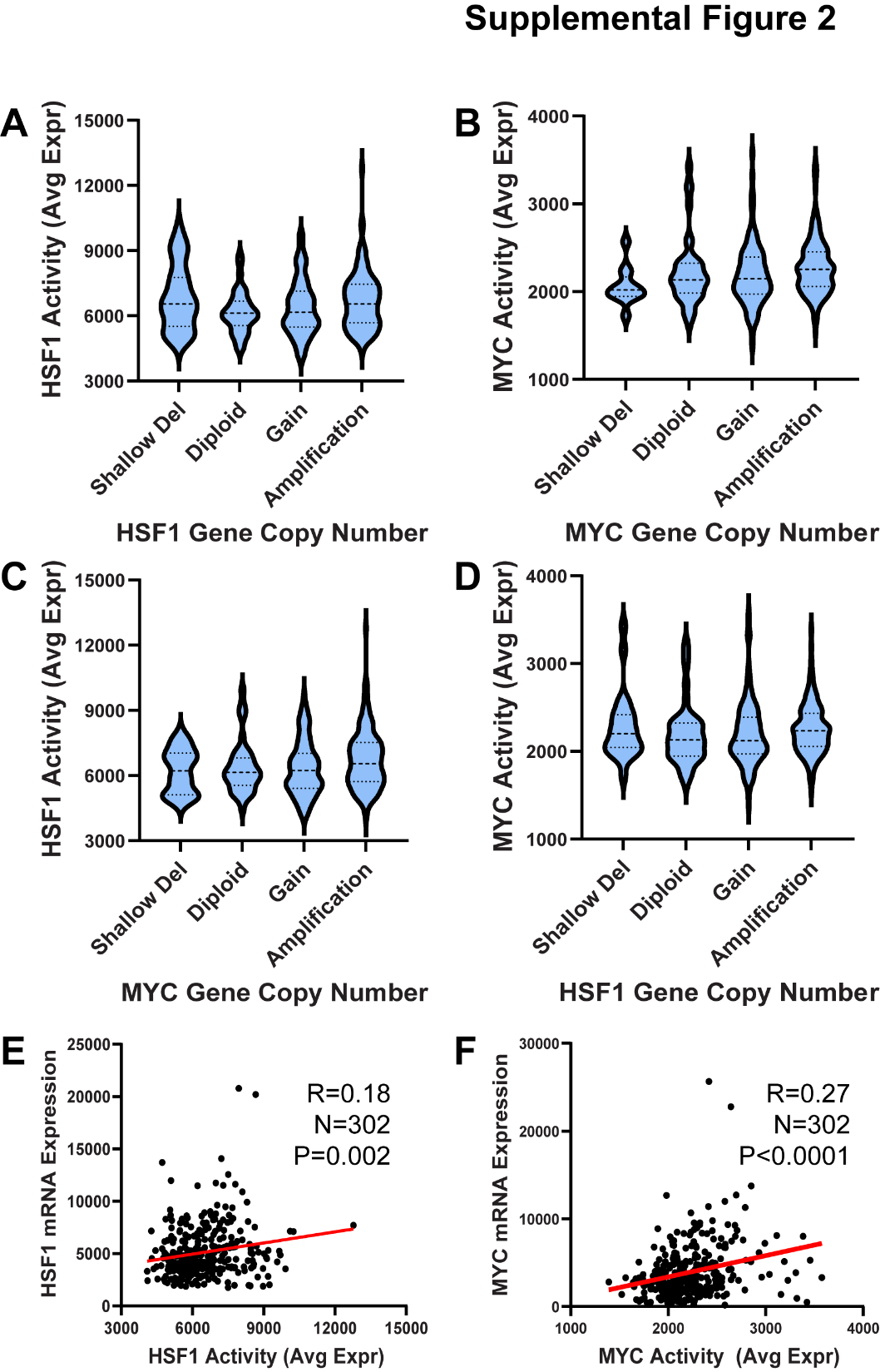
**

**Supplemental Figure 2: Effect of HSF1 or MYC copy number on their transcriptional activity.** MYC and HSF1 activity was calculated using published gene signatures in the TCGA-OV cohort. A-B) HSF1 activity (A) and MYC activity (B) are plotted by gene copy number of each respective gene. C-D) HSF1 activity (C) and MYC activity (D) are plotted by gene copy number of the opposite gene. E-F) Activity of HSF1 (E) and MYC (F) were correlated with expression of their respective mRNA.

**
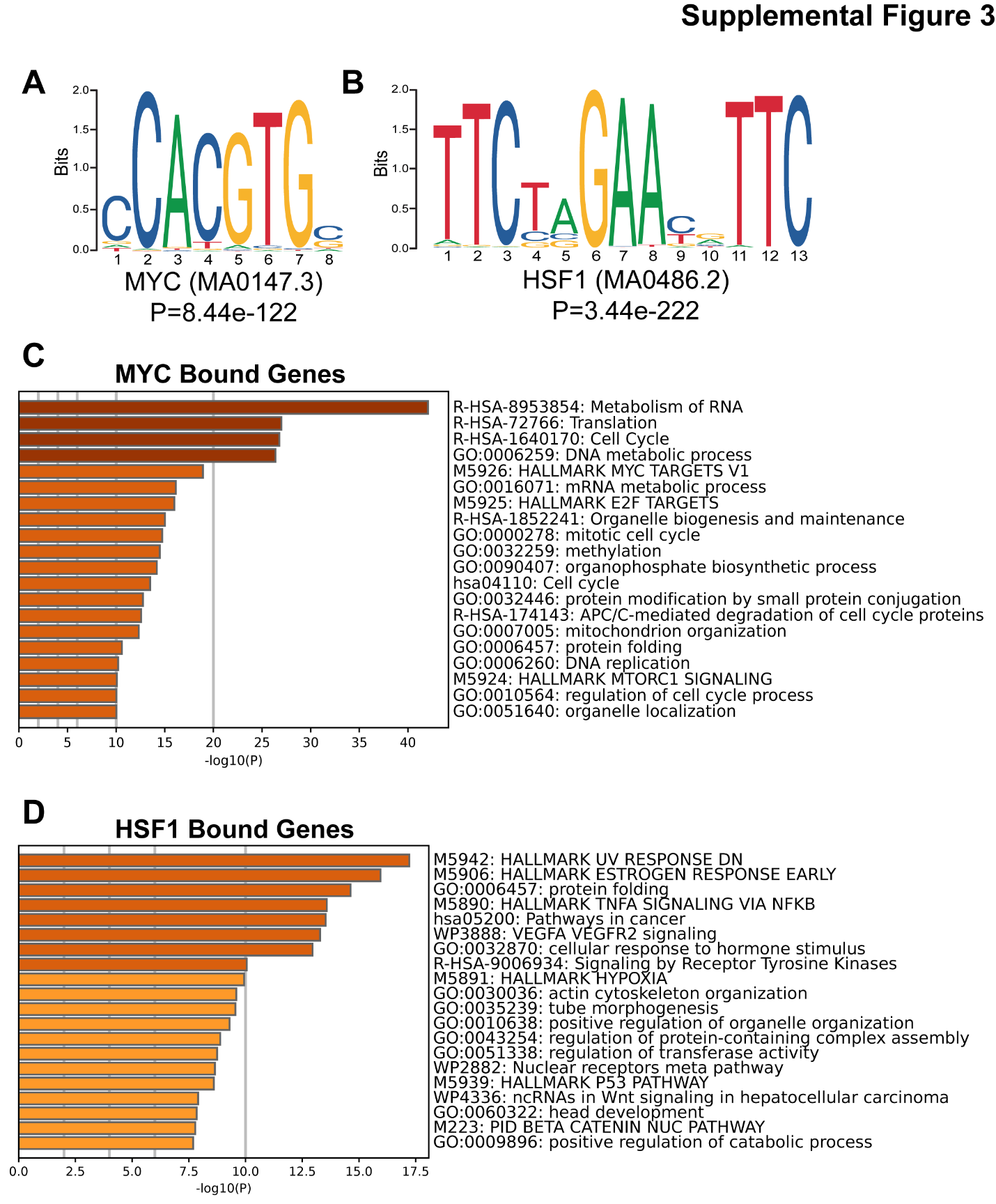
**

**Supplemental Figure 3: HSF1 and MYC CUT&RUN accurately detects their binding in HGSOC cells.** A) The MYC motif that was detected from the MYC CUT&RUN. B) The HSF1 motif that was detected from the HSF1 CUT&RUN. C-D) Gene ontology for annotated genes that were bound by MYC (C) and HSF1 (D).

**
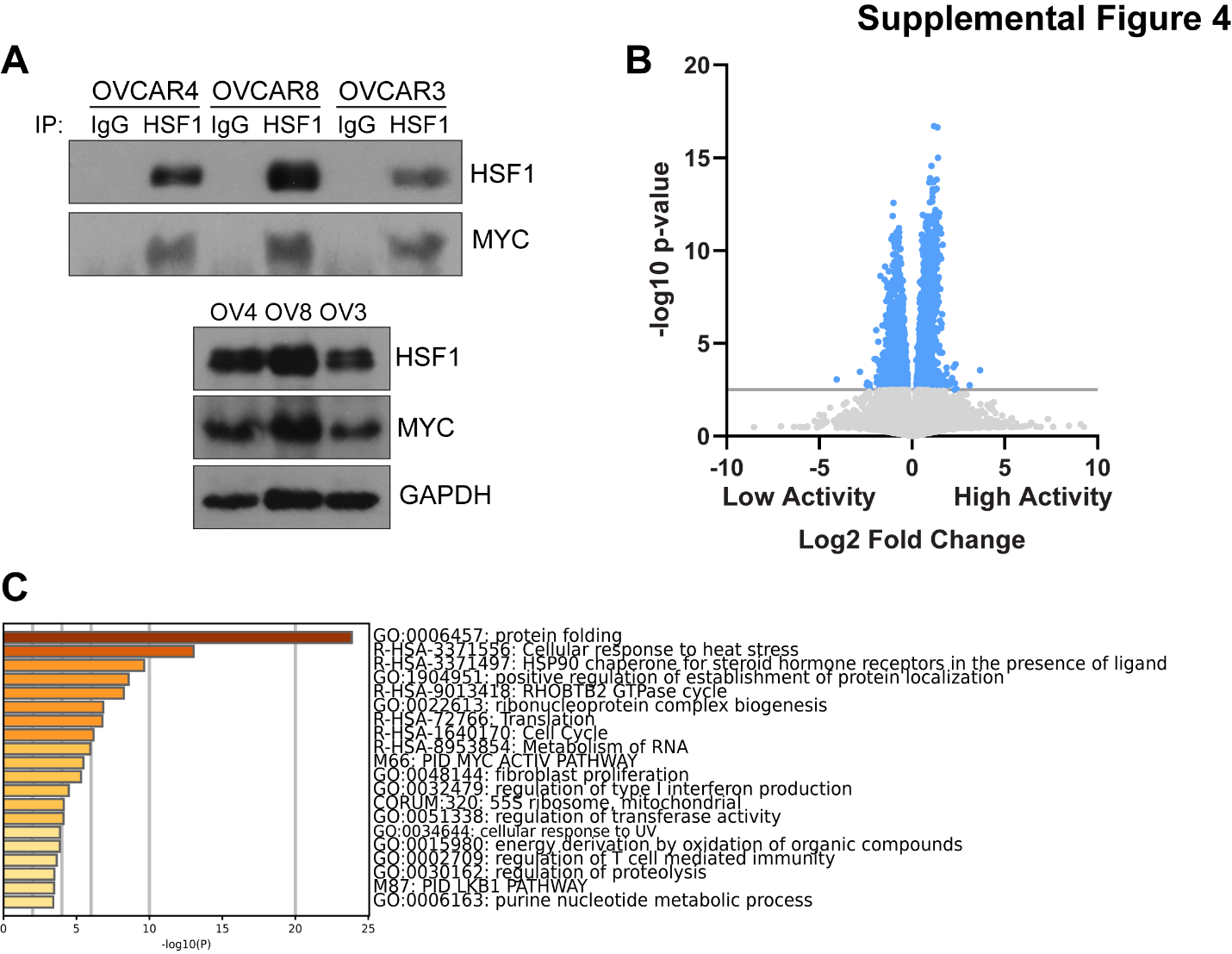
**

**Supplemental Figure 4: HSF1-MYC interaction and genes bound by MYC and HSF1 upregulated with high MYC and HSF1 activity.** A) Lysates from OVCAR 4 (OV4), OVCAR8 (OV8), and OVCAR3 (OV3) cells were subjected to immunoprecipitation for HSF1 and immunoblotted with the indicated antibodies. Lower panel is inputs immunoblotted with indicated antibodies. B-C) TCGA-OV samples were categorized into tertiles of MYC and HSF1 activity using published gene signatures. Samples that were in the highest tertile for both MYC and HSF1 activity were considered high activity for MYC and HSF1 whereas samples that were in the lowest tertile for both MYC and HSF1 activity were considered low activity for MYC and HSF1. A volcano plot visualizes the differential gene expression between samples with high or low MYC and HSF1 activity (B). There were 87 genes upregulated in samples with high MYC and HSF1 activity that were also co-bound by MYC and HSF1. These 87 genes were subjected to gene ontology analysis using Metascape (C).


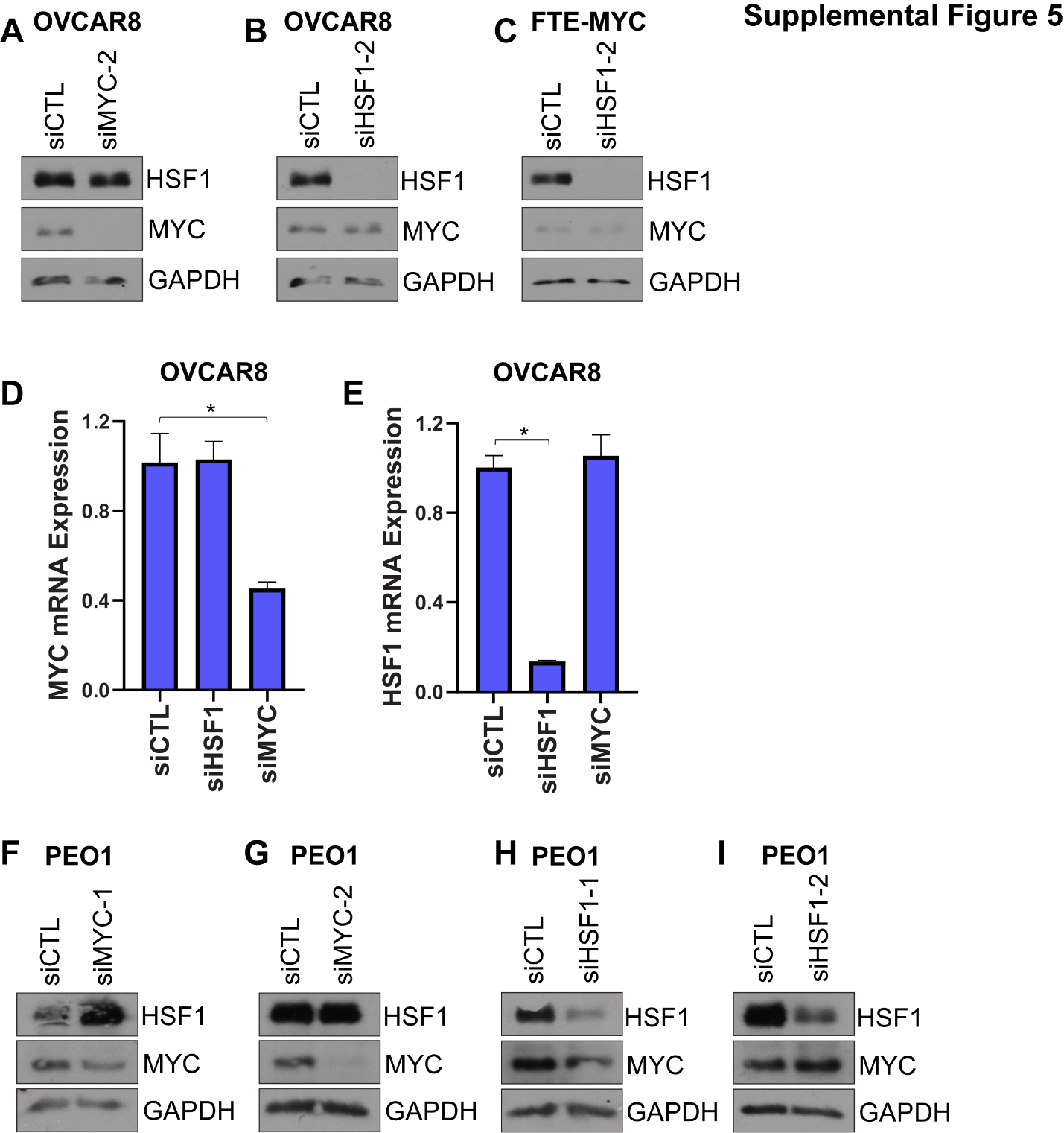


**Supplemental Figure 5: Cooperation between HSF1 and MYC expression in ovarian cancer cells.** A-B) OVCAR8 cells were transfected with control (CTL) siRNA or to a second MYC (A) or HSF1 (B) siRNA followed by immunoblotting of total protein with indicated antibodies. C) FTE-MYC cells were transfected with a second HSF1 siRNA followed by immunoblotting of total protein with indicated antibodies. D-E) OVCAR8 cells were transfected with either a control (CTL) siRNA or to a second MYC or HSF1 siRNA followed by RT-qPCR using total RNA with primer for MYC (D) or HSF1 (E). Significant differences were assessed using One-Way ANOVA with Tukey’s Post-hoc test. *Indicates significant difference (p<0.01). F-I) PEO1 cells were transfected with control (CTL) siRNA, MYC siRNA (F-G), or HSF1 siRNA (H-I). Total protein was subjected to immunoblotting with indicated antibodies.


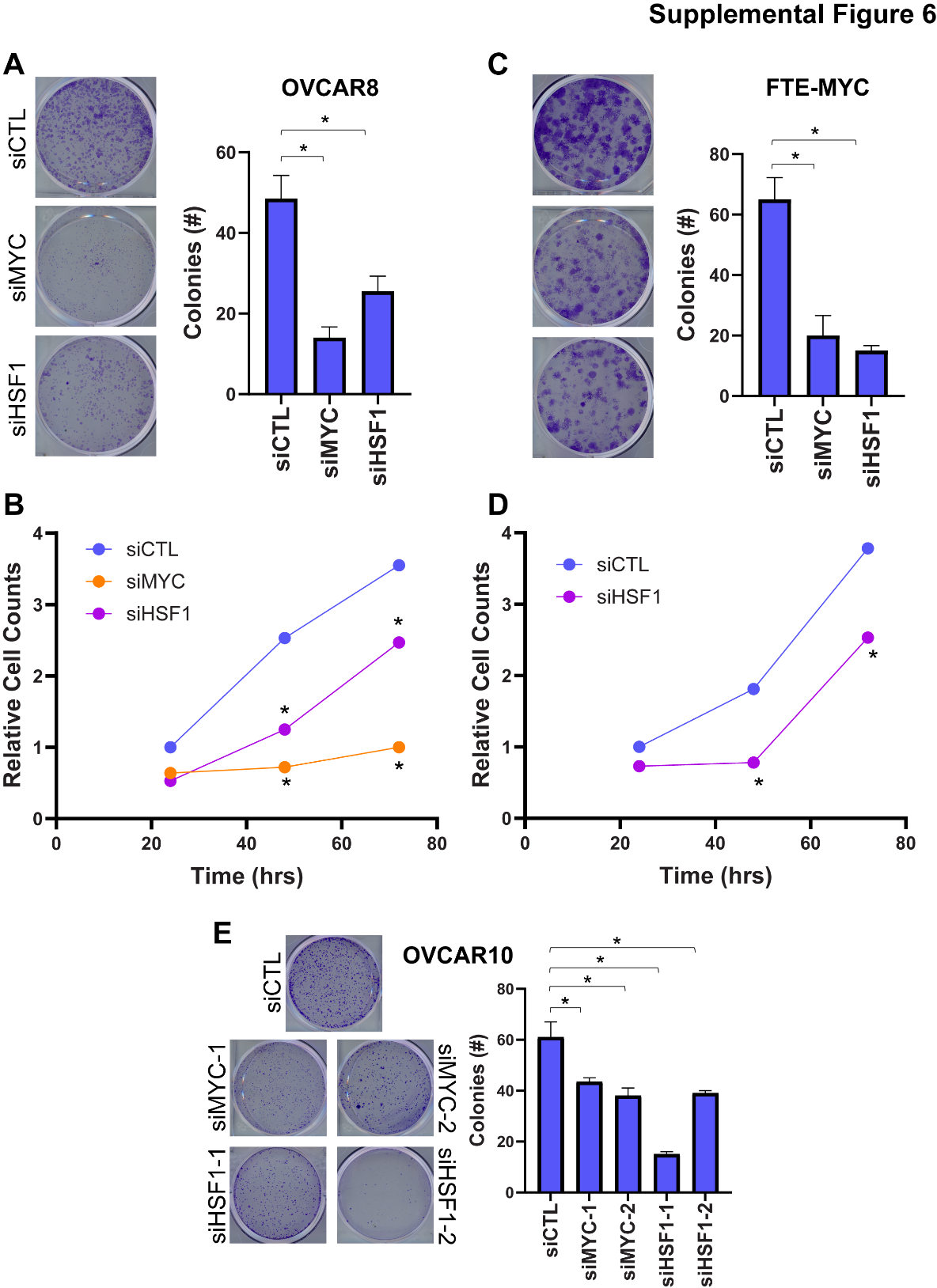


**Supplemental Figure 6: Knockdown of HSF1 or MYC with additional siRNAs has a greater effect in co-amplified cells compared to WT cells.** A-B) OVCAR8 cells were transfected with distinct siRNAs for 48 hrs and then subjected to a colony formation assay (A) and proliferation assay (B). C-D) FTE-MYC cells were transfected with distinct siRNAs for 48 hrs and then subjected to a colony formation assay (C) and proliferation assay (D). E) OVCAR10 cells were transfected with both sets of siRNAs for MYC or HSF1 and subjected to a colony assay. Colony assays were analyzed with one-way ANOVA and proliferation assays were analyzed with two-way ANOVAs, both with Tukey’s post-hoc test. *Indicates significant difference (p<0.01).


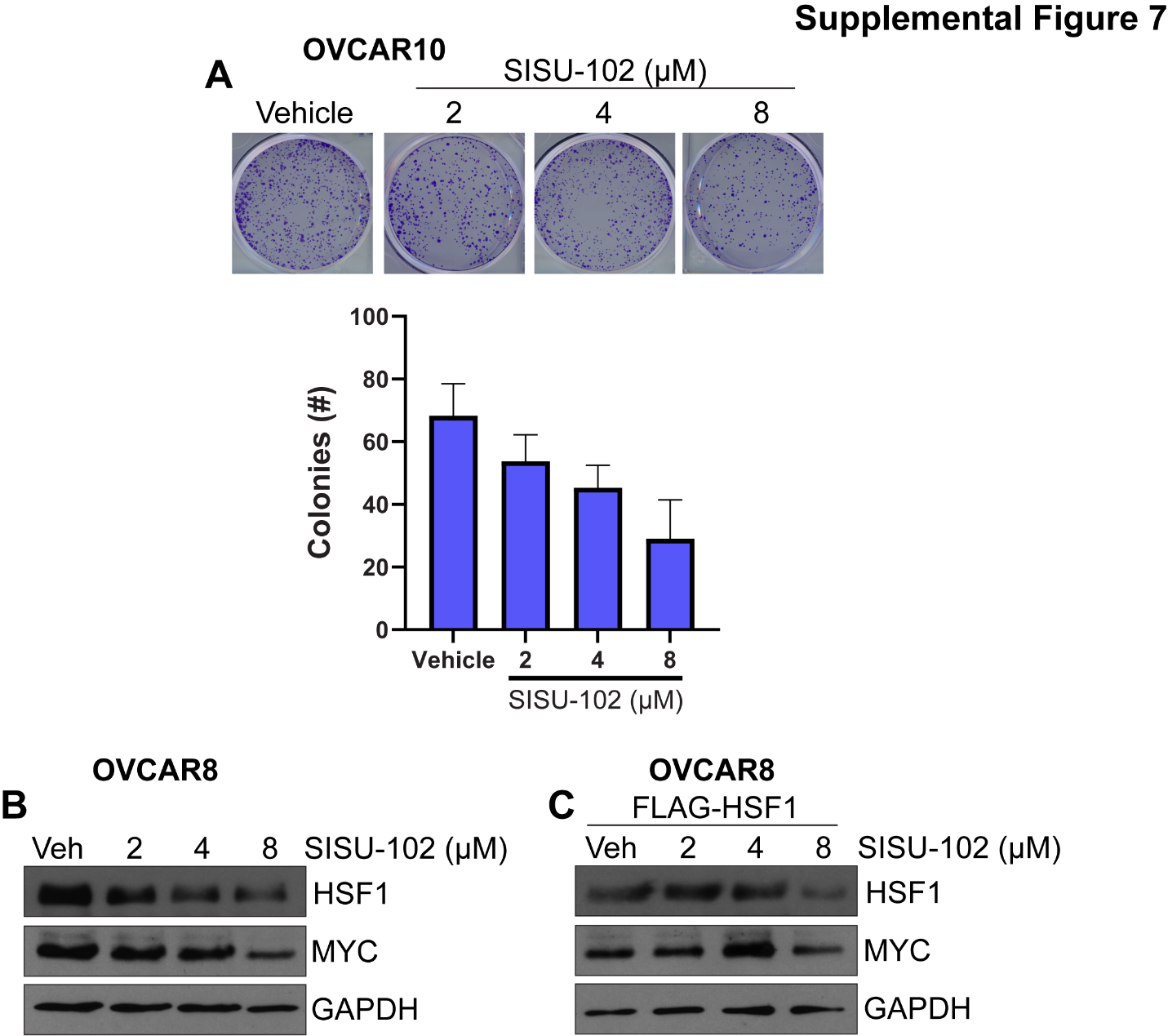


**Supplemental Figure 7: Effect of HSF1 inhibition on ovarian cancer cells.** A) OVCAR10 cells were subjected to a colony assay and were treated with the indicated doses of SISU-102. B-C) OVCAR8 cells were treated with SISU-102 for 24 hrs at the indicated doses either without (B) or with (C) HSF1 overexpression. Total protein was subjected to immunoblotting with the indicated antibodies.


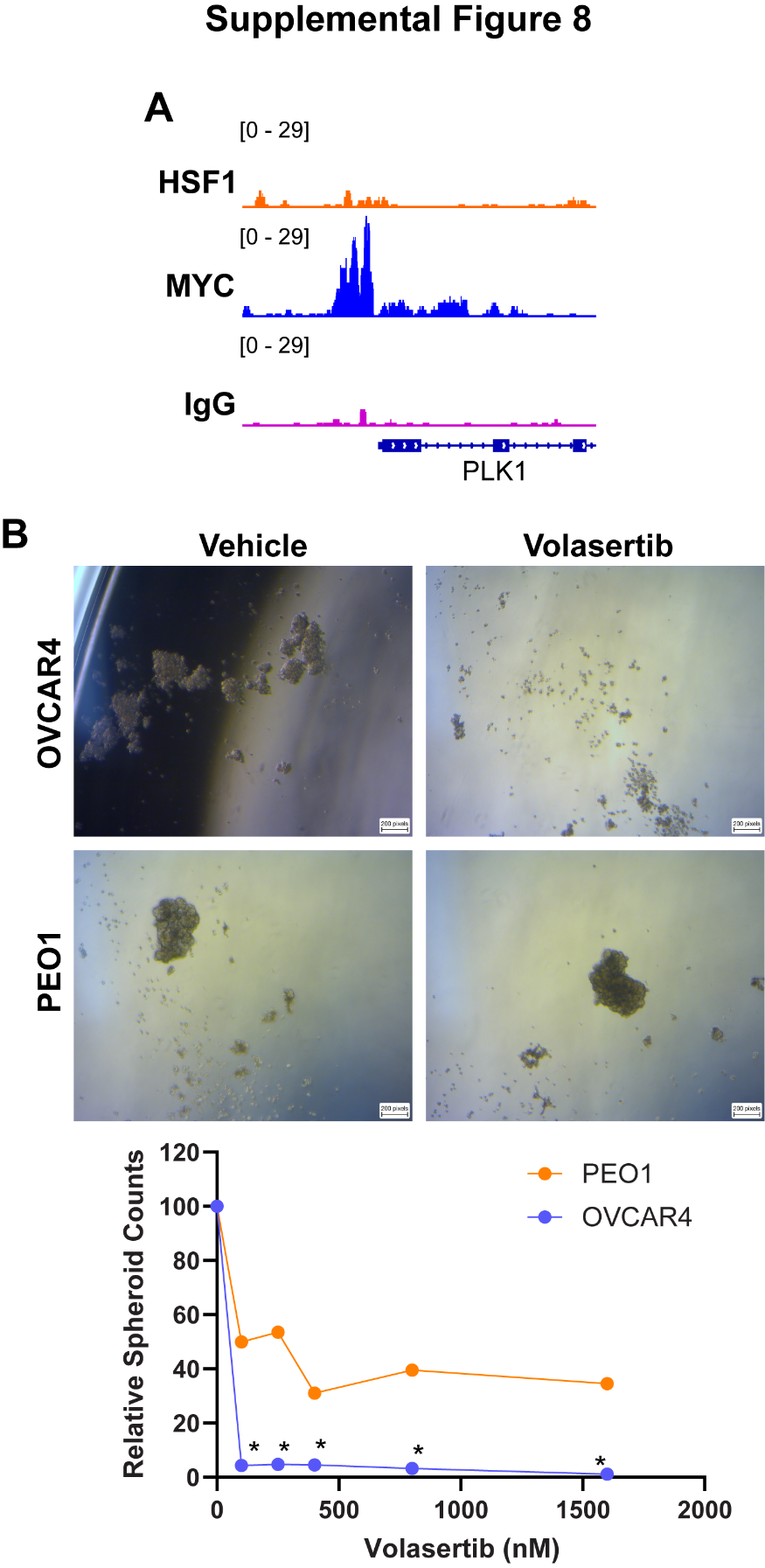


**Supplemental Figure 8: MYC binds the PLK1 gene promoter and effects of volasertib on spheroids.** A) Gene tracks HSF1 and MYC at the PLK1 promoter analyzed from CUT&RUN in Fig. 3. B) OVCAR4 and PEO1 cells were subjected to tumor spheroid growth for 12 days in the presence of vehicle or volasertib at the indicated doses.
