# Supplementary material for: MYC and HSF1 Cooperate to Drive Sensitivity to Polo-like Kinase 1 Inhibitor Volasertib in High-grade Serous Ovarian Cancer
Source: Cancer Res Commun. 2025 Feb 6;5(2):253–66. doi: 10.1158/2767-9764.CRC-24-0400 (PMC11799878; doi:10.1158/2767-9764.CRC-24-0400)
Supplement: Supplementary Tables — This document contains all Supplementary Tables. [file crc-24-0400_supplementary_tables_suppst.docx]

**Supplemental Tables**

**Supplemental Table 1: siRNA Sequences**

| **Gene Target** | **Sense Sequence** | **Antisense Sequence** | **Company** | **Catalog Number** |
| --- | --- | --- | --- | --- |
| HSF1 | CUGGUGCAGUCAAACCGGAtt | UCCGGUUUGACUGCACCAGtg | Bioneer | S6952 |
| HSF1 | CGUGUUCGACCAtt | GAACACGUGGAAGCtg | Bioneer | S6951 |
| HSF1 | GGACAAGAAUGAGCUCAGUtt | ACUGAGCUCAUUCUUGUCCag | Bioneer | S6950 |
| MYC | AGACCUUCAUCAAAAACAUtt | AUGUUUUUGAUGAAGGUCUcg | Bioneer | S9129 |
| MYC | GAGCUAAAACGGAGCUUUUtt | AAAAGCUCCGUUUUAGCUCgt | Bioneer | S9130 |
| MYC | ACAGCCCACUGGUCCUCAAtt | UUGAGGACCAGUGGGCUGUga | Bioneer | S9131 |
| HSF1 | CCAGUAUCCAAGAGCUCCU | AGGAGCUCUUGGAUACUGG | ThermoFisher | 3297-1 |
| HSF1 | GCGGCAGCUCAACAUGUAU | AUACAUGUUGAGCUGCCGC | ThermoFisher | 3297-2 |
| HSF1 | CACUUGGAUGCUAUGGACU | AGUCCAUAGCAUCCAAGUG | ThermoFisher | 3297-3 |
| MYC | AGAUGCUUCCUGGAGACUA | UAGUCUCCAGGAAGCAUCU | ThermoFisher | 4609-1 |
| MYC | CUCCUUGCAGCUGCUUAGA | UCUAAGCAGCUGCAAGGAG | ThermoFisher | 4609-2 |
| MYC | GAAAAGUGGAAGCGAGAUU | AAUCUCGCUUCCACUUUUC | ThermoFisher | 4609-3 |

**Supplemental Table 2: qPCR Primer Sequences**

| **Gene Target** | **Forward Primer (5’->3’)** | **Reverse Primer (5’->3’)** |
| --- | --- | --- |
| HSF1 | GCCTTCCTGACCAAGCTGT | GTCGAACACGTGGAAGCTGT |
| MYC | GCTGCCAAGAGGGTCA | CGCACAAGAGTTCCGTAG |
| HSP70 | CAAGATCACCATCACCAACG | TTCTTGGCTGACACCCTCTC |
| GAPDH | CCTGCACCACCAACTGCTTA | GGCCATCCACAGTCTTCTGAG |
